# PREVALENCE OF SENSORY IMPAIRMENTS VARY BY RACE IN OLDER US ADULTS

**DOI:** 10.1093/geroni/igac059.944

**Published:** 2022-12-20

**Authors:** Faaizah Arshad, Jayant Pinto, Kristine Yaffe, Willa Brenowitz

**Affiliations:** University of California, San Francisco, Los Angeles, California, United States; University of Chicago, Chicago, Illinois, United States; University of California, San Francisco, Los Angeles, California, United States; University of California, San Francisco, Los Angeles, California, United States

## Abstract

Few studies have examined racial/ethnic disparities in sensory function. We studied 3,005 US adults (aged 57-85, mean 69.3 years); 10% Hispanic, 17% Black, 71% White; National Social Life, Health, and Aging Project). Impairment was defined by established criteria for objectively measured vision, smell, taste, touch and interviewer-rated hearing. Vision (22%), hearing (19%), smell (23%), taste (15%), and touch (19%) loss were common. Hispanic and Blacks showed the highest prevalence of vision, smell, and touch dysfunction. Findings persisted after adjustment for age, sex, education, and cardiometabolic conditions. Blacks had higher odds of impaired vision (adjusted Odds Ratio [aOR]:1.61; 95%CI:1.12, 2.32), smell (aOR:2.64; 95%CI:1.81, 3.84)) and touch (aOR:1.81; 95%CI:1.23, 2.64)) compared to Whites. Hispanics had higher odds of impaired smell than Whites (aOR:2.33; 95%CI:1.47, 3.67). Racial/ethnic minorities face marked disparities in function of the classical senses. Understanding how these differences arise, including potential systemic/social mechanisms, may catalyze interventions that promote health equity.

